# Supplementary material for: Population health impact and economic evaluation of the CARDIO4Cities approach to improve urban hypertension management
Source: PLOS Glob Public Health. 2023 Apr 11;3(4):e0001480. doi: 10.1371/journal.pgph.0001480 (PMC10089359; doi:10.1371/journal.pgph.0001480)
Supplement: S1 Table — (DOCX) [file pgph.0001480.s002.docx]

|  | **Quality of Care** | **Ensure Access** | **Policy Reform** | **Data and digital** | **Intersectoral partnership** | **Local Ownership** |
| --- | --- | --- | --- | --- | --- | --- |
| **São Paulo** | Develop a new, **standardized NCD management protocol** jointly with the City Hall, incl. algorithm of prevention and care  Capacity building for clinic managers through **management trainings and performance management system**  **Training of health providers and multi-disciplinary teams** on hypertension and CVD management through online continuous medical education.  Support strengthening the **role of pharmacists** in supporting treatment and treatment adherence. | Active screening and acceleration of early detection through the **implementation of BP self-screening corners** (in-clinic).  **Distribution of information material** promoting BP screening in all clinics in the province of Itaquera.  **Community engagement and mobilization** for opportunistic and out-of-office screening. | Introduce **targets for chronic disease management** in primary care centers and district supervision teams.  Emphasize **cardiovascular risk scores** and **data quality.**  Support task-sharing and task-shifting initiatives through health-care workers | **Strengthen hypertension** outcome and output **data collection** and include in monitoring activities.  Reinforce and train **primary care management with data**.  Introduce solutions to monitor **district coverage and targets**, **track numbers of hypertensive** patients. | **School-based program** aimed at raising **awareness on NCDs** (CEU – Amigo do Coração), incl. training of students and professional educational leaders on NCD management, risk factors, nutrition, physical education, and heart health literacy.  Build **partnerships with community champions** (e.g. football and samba clubs) to bring awareness and opportunistic screening possibilities. | **Leadership through the City Health Authorities** to establish coordinated care for chronic patients.  Establish **co-creation process in the design, optimization, and scale** of interventions. |
| **Dakar** | **Standardized hypertension management** with clinical decision support for health providers and trainings.  Strengthen hypertension **referral systems to secondary and tertiary healthcare levels**. | **Systematic screening** of all adults >18 presenting in the city’s primary health centres.  **Community health awareness campaigns** with community activities.  **Introduction of task-shifting** to increase overall coverage and efficiency of primary health services. | Reinforce the routine calculation of **cardiovascular risk scores.** | **Enhanced hypertension data collection.** First consolidated paper-based hypertension registry to collect and monitor all hypertension data on district level in primary care. | Establish public private partnerships on **workplace and school programs** in revising the national curriculum for primary and secondary schools to integrate information on key CVD and NCD risk factors.  **Agricultural sector.** Public-private partners from the agricultural sector collaborated to increase the availability of fresh foods in the city.  Include medical societies to strengthen decision-making bodies around hypertension and NCDs management **policy decision-making** | **Ministry of Health leadership** through a multisector committee to address NCD and the joint development of the national strategy to address NCDs. |
| **Ulaanbaatar** | **Up dated and standardized hypertension guidelines** and their translation into simplified algorithms.  **Continued medical education for multi-disciplinary team** (HCP, nurses, clinic managers, social workers etc.) on standardized management of high BP. | **Proactive hypertension screening** and focus on **early detection** in primary health centres.  New component on **engagement of pharmacists in hypertension detection** and care with training and tools for adequate measurement and health information delivery. | Advocated for **health policy reform** e.g. primary care budget, dietary improvement strategies, tobacco taxes, etc.  Introduce **hypertension cascade indicators** and tracking of patient numbers in primary care.  **Cardiovascular risk scores** were routinely calculated and included into the evaluation of hypertensive patients. | Inform enhanced **data-collection mechanisms** for hypertension carried forward by the Ministry of Health and National Health Insurance. | **Health workplace initiatives** and introduced **physical and nutritional education in** schools, supporting to develop a curriculum for schools on information on NCD prevention.  Develop **early detection model** with community partners and implementing referral system.  Structure patient support and advocacy groups. | The Ministry of Health involvement in establishing **steering committees** focused on broad strategic direction, which included the city mayor and senior public managers with responsibilities for relevant sectors, including health, education, IT and communication, and agriculture. |

HCP: Health care professional; BP: blood pressure; NCD: non-communicable diseases; IT: information technology; CVD: cardiovascular disease

***Table S1: Summary of CARDIO activities by city.***
