# Supplementary material for: Population health impact and economic evaluation of the CARDIO4Cities approach to improve urban hypertension management
Source: PLOS Glob Public Health. 2023 Apr 11;3(4):e0001480. doi: 10.1371/journal.pgph.0001480 (PMC10089359; doi:10.1371/journal.pgph.0001480)
Supplement: S1 Text — Table A in S1 Text: Assumed CVD risk by sex and CVD risk category. Table B in S1 Text: Patient distribution across sex and CVD risk category for each location and scenario. Table C in S1 Text: Initial blood pressure of patients who achieved BP control under the ‘CARDIO’ and ‘NO CARDIO’ scenarios. Table D in S1 Text. Model Assumptions and parameters. (DOCX) [file pgph.0001480.s003.docx]

## **S1 Text: Modelling methodology**

**Individual-level risk of developing CVD within 10 years for patients with uncontrolled hypertension**

During the patients’ medical assessment, their CV risk was evaluated by physicians and categorized as low, medium, or high (see [1], S1 Table). For our model, the risk of having any CV event for patients with uncontrolled hypertension in each category was quantified using the Framingham Risk Score (FRS) [2]. We extracted individual-level 10-year CVD risks, $FRS_{s,c}$, where $s$ is the individual’s sex and $c$ their CVD risk category. As the FRS considers a point-based stratification within each risk category and this information was not available for our study, the mid-value for each risk category was used (Table A).

| Sex (s) | | Male | | | Female | | |
| --- | --- | --- | --- | --- | --- | --- | --- |
| Risk category (c) | | Low | Medium | High | Low | Medium | High |
| Framingham Risk Score | |  |  |  |  |  |  |
|  | Lower bound | 0.00% | 11.20% | 21.60% | 0.00% | 10.00% | 21.50% |
|  | Upper bound | 9.00% | 18.40% | 30.00% | 9.00% | 18.51% | 30.00% |
|  | Mid-value ($FRS_{s,c}$) | **4.7%** | **14.8%** | **25.8%** | **4.3%** | **14.26%** | **25.75%** |

***Table A: Assumed CVD risk by sex and CVD risk category****.*

**Inferring population-level CVD rates**

For each location, $l$, CVD event rates across the treated patient population were calculated. For each city, the distribution of treated patients across CVD risk categories by sex at baseline (No CARDIO scenario) and at the end of the reporting period (CARDIO scenario) for each city were extracted from the literature [1] (Table B). Only patients with complete information were included. At baseline, 12% of males and 9% of females had missing data for CV risk in Dakar, and 86.5% and 86.1% in São Paulo. For Ulaanbaatar the distribution at baseline was inferred from the BP and risk distribution in the last quarter, as no direct data was available. In addition, 0.3% % in Ulaanbaatar had missing information for sex, 2.0% in Dakar, and 4.5% in São Paulo.

|  |  | **No CARDIO (baseline quarter)** | | **CARDIO (last quarter)** | |
| --- | --- | --- | --- | --- | --- |
| **Ulaanbaatar** (n=10,074; 37% male) | | | | | |
|  | **CVD risk** | **- Male** | **Female** | **Male** | **Female** |
|  | low | 46% | 54% | 46% | 54% |
|  | medium | 43% | 40% | 43% | 40% |
|  | high | 11% | 6% | 11% | 6% |
| **Dakar** (n=5,235; 22% male) | | | | | |
|  | **CVD risk** | **- Male** | **Female** | **Male** | **Female** |
|  | low | 17% | 21% | 19% | 22% |
|  | medium | 31% | 30% | 31% | 30% |
|  | high | 52% | 48% | 50% | 48% |
| **São Paulo** (n=5,844; 27% male) | | | | | |
|  | **CVD risk** | **- Male** | **Female** | **Male** | **Female** |
|  | low | 13% | 14% | 18% | 15% |
|  | medium | 10% | 22% | 18% | 21% |
|  | high | 77% | 64% | 65% | 64% |

***Table B: Patient distribution across sex and CVD risk category for each location and scenario****.*

**For Ulaanbaatar, patient risk profiles at baseline were not available. We therefore extrapolated the risk distribution at baseline by backwards inference from last quarter, assuming that the change in risk distribution between the two quarters was similar to the changes observed in Dakar*

Based on distribution of patients across risk categories, the average 10-year CVD risk for any patient of sex s ($s=m$ for male and $s=f$ for female) in location $l$, $r_{s,l}$, was:

$$r_{s,l}=\sum_{c} p_{s,c,l} FRS_{s,c}$$

where $p_{s,c,l}$ is the proportion of patients assigned to CVD category $c$ among all patients of sex $s$ in location $l$, and $FRS_{s,c}$ the FRS for patients with sex $s$ in category $c$, extracted from the literature [2].

The average 10-year CVD risk for any patient in location $l$, $R_{l}$, was therefore:

$$R_{l}= m_{l} r_{m,l}+\left( 1-m_{l} \right)r_{f,l}$$

where $m_{l}$ is the proportion of males in location $l$, $r_{m,l}$ is the average 10-year CVD risk for a male in location $l$ and $r_{f,l}$ is the average 10-year CVD risk for a female in location $l$.

From the individual-level 10-year risk, we extrapolated annual average CVD event rates, $S$, for uncontrolled hypertension patients for each location $l$:

$$S_{l}= 1-\left( 1-R_{l} \right)^{1/10}$$

The estimated total number of annual CVD events for each location in a population of exclusively uncontrolled patients was therefore $C_{l}={S_{l}N}_{l}$, where $N_{l}$ is total number of patients treated in each city, derived from the literature [1]. In the absence of country-specific rates, it was assumed that 30% of CVD events would be strokes [3].

Patients who achieved hypertension control were assumed to have a decreased risk of CVD events and mortality. Risk reductions for controlled patient groups extracted from the literature [4], and applied to the baseline CVD event rate in each location, $C_{l}$. For Ulaanbaatar, the control rate for the ‘No CARDIO’ scenario (at baseline, Q1 2018) was 3.1%, and 19.7% for the ‘CARDIO’ scenario (final quarter, Q3 2019). For Dakar, control rates were 13% for (Q1 2019) `CARDIO (Q4 2019), respectively, and for São Paulo, 12.3% (Q4 2018) and 31.2% (Q4 2019), respectively. For patients who achieved BP control with prior BP, $b$, a deduction equivalent to the complement of the respective hazard ratio was applied. The hazard ratios and number of individuals in each category for each city at baseline are summarized in Table C.

|  |  | **Initital BP** | | | | | |
| --- | --- | --- | --- | --- | --- | --- | --- |
|  |  | <120 and <80 | 120-129 or 80-84 | 130 - 139 or 85 - 89 | 140 - 159 or 90 - 99 | 160 - 179 or 100 - 109 | ≥ 180 or ≥ 110 |
| **HR after control** | | | | | | | |
|  | CHD ($h_{b}$) | 1.00 | 1.00 | 0.96 | 0.83 | 0.51 | 0.51 |
|  | Stroke ($t_{b}$) | 1.00 | 1.00 | 1.00 | 0.74 | 0.39 | 0.39 |
| **Number of patients at baseline** ($n_{b,l})$ | | |  |  |  |  |  |
|  | Ulaanbaatar | 103 | 208 | 2358 | 4012 | 2538 | 857 |
|  | Dakar | 94 | 200 | 404 | 2057 | 1424 | 1057 |
|  | São Paulo | 148 | 341 | 229 | 2480 | 1972 | 675 |

***Table C: Initial blood pressure of patients who achieved BP control under the ‘CARDIO’ and ‘NO CARDIO’ scenarios***

The annual number of CHD events per year in location $l$ was therefore

$$H_{l}=0.7\left( C_{l}- \sum_{b} n_{b,l}\left( 1-h_{b} \right) \right),$$

where $n_{b,l}$ is the number of patients in location $l$, who achieved control after having BP category $b$ at enrolment, and $h_{b}$is the hazard ratio for incurring a CHD event for a patient with controlled hypertension and an initial BP of $b$.

Similarly, the annual number of strokes in each location was described as:

$$T_{l}=0.3\left( C_{l}- \sum_{b} n_{b,l}\left( 1-t_{b} \right) \right),$$

where $t_{b}$ is the hazard ratio for incurring a stroke for a patient with controlled hypertension and initial an BP of $b$.

Outcomes were modelled over two years. The resulting populations were used as baseline for the subsequent extended time model.

## **Model Assumptions and parameters**

Table D describes the assumptions and parameters used in the models

|  | **São Paulo** | **Dakar** | **Ulaanbaatar** | **Definition, use, and references** |
| --- | --- | --- | --- | --- |
| **Population characteristics** | | | | |
| Total population treated | 5844 | 5236 | 10075 | Raw data [1] |
| Mean age | 62 | 58 | 61 | Mean age was calculated based on proportion of patients in each age category (mid-point value) in the last quarter of CARDIO implementation. Mean age was used to derive mortality. [1] |
| Male proportion | 27% | 22% | 37% | Raw data [1] based on cumulative total of patients prescribed medication treatment (patients with at least one visit during February 2018– September 2019 for Ulaanbaatar, April 2018 - December 2019 for Dakar, and October 2018 – December 2019 for São Paulo). The sex ratio was used to derive sex-adjusted population-level CHD and stroke rates. |
| **Mortality** | | | | |
| Mortality multiplier for controlled hypertension | 2.10 | 2.10 | 2.10 | [5], adjusted with age specific mortality (life table : <https://apps.who.int/gho/data/view.main.61450?lang=en> |
| Mortality multiplier for uncontrolled hypertension | 6.10 | 6.10 | 6.10 | [5], adjusted with age specific mortality (life table : <https://apps.who.int/gho/data/view.main.61450?lang=en> |
| **Disease** | | | | |
| Average patient-level annual risk of CVD | Usual care: 2.29%  CARDIO: 2.22% | Usual care: 1.96%  CARDIO: 1.94% | Usual care: 1.08%  CARDIO: 1.07% | Annual Risk of CVD includes CHD, stroke, heart failure, and CVD deaths |
| Proportion of CVD events that are strokes | 30% | 30% | 30% | [3]. Due to additional comorbidity risk factor (diabetes, obesity etc.) and conservative assumptions we rounded the stroke events to 30% instead of 33%. |
| **Event costs** | | | | |
| Controlled hypertension, yearly | US$191 | US$139 | US$34 | Annual healthcare costs per person with hypertension (including averages medicines cost, health insurance, medical consultation, laboratory tests, transportation, and meals) [6] |
| Uncontrolled hypertension, yearly | US$191 | US$139 | US$34 |  |
| Coronary heart disease event | US$1522 | US$1111 | US$273 | Cost of a coronary heart disease event (including outpatient and hospitalization costs) [7] |
| Stroke event | US$5864 | US$4280 | US$1053 | [8] [9] |
| Annual loss of productivity per person with hypertension | US$153 | US$112 | US$27 | Following partial or total absence from work; estimated via proportional per-capita income for one working day [6], adjusted to inflation |
| Purchasing power parity factor | 1 | 1.37 | 5.57 | World Bank’s International Comparison Program (ICP; ). Baseline = São Paulo. |
| Discounting rates | 5% | 3% | 3% | Brazil Ministry of Health, WHO Choice Methodology |
| **Utility of health states** | | | | |
| *Hypertension utility with age* | | | | |
| Age 50-59 | 0.84 | 0.84 | 0.84 | [10] |
| Age 60-69 | 0.82 | 0.82 | 0.82 | [10] |
| Age 70-79 | 0.78 | 0.78 | 0.78 | [10] |
| Age 80-100 | 0.74 | 0.74 | 0.74 | [10] |
| *Annual disutility* | | | | |
| Coronary disease | -0.018 | -0.018 | -0.018 | [10] |
| Stroke | -0.048 | -0.048 | -0.048 | [10] |

***Table D. Model Assumptions and parameters***

1. Boch J, Venkitachalam L, Santana A, Jones O, Reiker T, Rosiers SD, et al. Implementing a multisector public-private partnership to improve urban hypertension care in low-and middle- income countries. BMC Public Health. 2022;22(2379).

2. Wilson PW, D’Agostino RB, Levy D, Belanger AM, Silbershatz H, Kannel WB. Prediction of coronary heart disease using risk factor categories. Circulation. 1998;97(18):1837-47.

3. Group L-TIwPiIDS. Prevention of cardiovascular events and death with pravastatin in patients with coronary heart disease and a broad range of initial cholesterol levels. New England Journal of Medicine. 1998;339(19):1349-57.

4. Bundy JD, Li C, Stuchlik P, Bu X, Kelly TN, Mills KT, et al. Systolic blood pressure reduction and risk of cardiovascular disease and mortality: a systematic review and network meta-analysis. JAMA cardiology. 2017;2(7):775-81.

5. da Silva TLN, Klein CH, da Rocha Nogueira A, Salis LHA, e Silva NAdS, Bloch KV. Cardiovascular mortality among a cohort of hypertensive and normotensives in Rio de Janeiro-Brazil-1991–2009. BMC Public Health. 2015;15(1):1-11.

6. da Costa JSD, Fuchs SC, Olinto MTA, Gigante DP, Menezes AMB, Macedo S, et al. Cost-effectiveness of hypertension treatment: a population-based study. São Paulo Medical Journal. 2002;120:100-4.

7. Schlatter RP, Hirakata VN, Polanczyk CA. Estimating the direct costs of ischemic heart disease: evidence from a teaching hospital in BRAZIL, a retrospective cohort study. BMC cardiovascular disorders. 2017;17(1):1-11.

8. Dos Santos R, Alves F, Urbich M, Villa G, Farsky P. Budget Impact Analysis Of Adopting Evolocumab In The Brazilian Private Healthcare System For Patients With Uncontrolled LDL-C And High Cardiovascular Risk. Value in Health. 2017;20(9):A914-A5.

9. de Souza CPR, Santoni NB, de Melo TG, de Oliveira Figueiredo MJ, da Costa Darrieux FC, Piegas LS, et al. Cost-effectiveness and cost-utility analyses of dabigatran compared with warfarin in patients with nonvalvular atrial fibrillation and risk factors for stroke and systemic embolism within Brazilian private and public health care systems perspectives. Value in health regional issues. 2015;8:36-42.

10. Mould-Quevedo JF, Gutiérrez-Ardila MV, Molina JEO, Pinsky B, Zea NV. Cost-effectiveness analysis of atorvastatin versus rosuvastatin in primary and secondary cardiovascular prevention populations in Brazil and Columbia. Value in health regional issues. 2014;5:48-57.
