# Supplementary material for: Population health impact and economic evaluation of the CARDIO4Cities approach to improve urban hypertension management
Source: PLOS Glob Public Health. 2023 Apr 11;3(4):e0001480. doi: 10.1371/journal.pgph.0001480 (PMC10089359; doi:10.1371/journal.pgph.0001480)
Supplement: S2 Text — Table A in S2 Text: Phases of work in the three cities. Table B in S2 Text: Assumed implementation cost for the CARDIO approach in Ulaanbaatar, Dakar, and São Paulo. (DOCX) [file pgph.0001480.s004.docx]

### **S2 Text: Cost assumptions**

Data collection in Ulaanbaatar, Dakar and São Paulo started in quarter Q1, Q2 and Q4 of 2018 respectively, resulting in reporting periods of 21, 12, and 15 months of implementation.

The implementation period in each city was organized into distinct but fluid phases of groundwork, design and intervention (summarized in Table A). The groundwork phase consisted of establishing contact and agreements with government entities, feasibility analysis, stakeholder engagement and first technical workshops, and tender processes to identify the in-country implementation partner to support the initiative. The design phase consisted of desk research, co-creation workshops with local authorities, health care providers and patients, field research and interviews, user journey formulation and systemization of solutions. The execution phase consisted of the formal launch of the planned interventions in the target districts (in some cities such as Dakar, the roll-out was phased).

Budget spend was determined through the extraction of yearly financial information internal to the Novartis Foundation (i.e. spending per partner) and was adjusted to the number of months required for the formal intervention phase. Budget spend per city considers Novartis Foundation funding to in-country implementation partner, data partner and local health authorities only. Funding for the global evaluation of the initiative was excluded from the analysis as it relates to the evaluation of health outcomes for cross-country comparison.

A 15% discount was applied to account for overhead spend of in-country partners (administrative fees). To reflect the actual budget a government would require to implement the program with local resources, an additional 50% discount was applied to the budget minus overheads to account for the investments required build the partnership ecosystem that would implement the approach. Such ecosystem building activities included stakeholder engagement, preparation of updated data collection systems, and the onboarding of local authorities and actors. This amount was required to bring the ecosystem to a functional state for basis data collection and implementation and to account for initial local learnings.

| Phases | Ulaanbaatar | Dakar | São Paulo |
| --- | --- | --- | --- |
| Groundwork |  | Jan 2017-July 2017 | June 2017- Feb 2018 |
| Design | Sept 2017-Feb 2018 | July 2017-March 2018 | Feb 2018-Aug 2018 |
| Intervention | Feb 2018–Sep 2019 | West: Apr 2018–Dec 2019  Center: July 2018–Dec 2019  North: July 2018–Dec 2019  South: Jan 2019–Dec 2019 | Oct 2018-Dec 2019 |

***Table A. Phases of work in the three cities***

The resulting assumed costs in the three cities are summarized in Table B.

|  | Ulaanbaatar | Dakar | São Paulo |
| --- | --- | --- | --- |
| Intervention period | Feb 2018 – Sep 2019 | April 2018 – Dec 2018**  Jan 2019 – Dec 2019*** | Oct 2018 – Dec 2019 |
| Period of intervention (months) | 21 | 12 | 15 |
| In-country implementation budget spend (USD)* | 1,561,814.77 | 523,257.96**  1,009,239.54*** | 1,391,127.95 |
| In-country partner overhead discount (15%) (USD) | 234,272 | 151,386 | 208,669 |
| Budget minus overhead (USD) | 1,327,543 | 857,854 | 1,182,459 |
| Preparatory activities discount (50%) (USD) | 663,771 | 428,927 | 591,229 |
| **Final implementation budget for modelling (USD)** | **663,771.28** | **428,926.80** | **591,229.38** |

*2018-19 budget spend accounts for funding to in-country implementation partner, data partner and local health authorities.

** Dakar budget referring to Q2 2018-Q4 2018 and introduction of interventions in the West, Center and North district, period was disregarded due to gaps in data points

*** Dakar budget referring to Q1 2019-Q4 2019 considering all 4 onboarding districts, considered for the modelling

***Table B. Assumed implementation cost for the CARDIO approach in Ulaanbaatar, Dakar, and São Paulo***
