# Supplementary material for: Population health impact and economic evaluation of the CARDIO4Cities approach to improve urban hypertension management
Source: PLOS Glob Public Health. 2023 Apr 11;3(4):e0001480. doi: 10.1371/journal.pgph.0001480 (PMC10089359; doi:10.1371/journal.pgph.0001480)
Supplement: S3 Text — Table A in S3 Text: Estimated CHD events, strokes, deaths, and events averted following implementation of the CARDIO approach in, Dakar under alternative assumptions for a 2-year implementation phase and a 10-year projection phase. Event projections for “No CARDIO” assume control rates consistent with baseline control rates (7%). “CARDIO” assumes control rates equivalent to those measured in the last quarter of CARDIO implementation (19%). Control rates were assumed to remain consistent throughout the projection period. Table B in S3 Text: The incremental cost summarizes the difference in total costs (program cost and CV-event costs) for No CARDIO vs CARDIO case. +Estimates extracted from [36]. The thresholds are adjusted for PPP and in-country opportunity costs. *Estimates extracted from [38]. $WHO CHOICE stipulates a cost-effectiveness threshold of three times the per-capita GDP. (DOCX) [file pgph.0001480.s005.docx]

### **S3 Text: Alternative simulation for Dakar**

To account for the increase in hypertension control rate during the expansion period in Dakar, we conducted an exploratory analysis, using the baseline control rate from Q2 2018 (7%) and baseline population characteristics from Q1 2019 and considering 18 months of implementation (Q2 2018-Q42019). The results of this analysis on health outcomes are summarized in Table A and the health economic analysis is summarized in Table B

|  | Dakar (n=5236) | | |
| --- | --- | --- | --- |
|  | No CARDIO | CARDIO | Averted |
| **Implementation phase** |  |  |  |
| ***CHD*** |  |  |  |
| Total | 169,0 | 154,0 | 15,0 |
| Per 1,000 patients | 32,3 | 29,4 | 2,9 |
| ***Stroke*** |  |  |  |
| Total | 51,0 | 46,0 | 5,0 |
| Per 1,000 patients | 9,7 | 8,8 | 1,0 |
| ***Deaths*** |  |  |  |
| Total | 107,0 | 107,0 | 0,0 |
| Per 1,000 patients | 20,4 | 20,4 | 0,0 |
|  |  |  |  |
| **10-year projection phase** |  |  |  |
| ***CHD*** |  |  |  |
| Total | 873,0 | 830,0 | 43,0 |
| Per 1,000 patients | 166,7 | 158,5 | 8,2 |
| ***Stroke*** |  |  |  |
| Total | 262,0 | 246,0 | 16,0 |
| Per 1,000 patients | 50,0 | 47,0 | 3,1 |
| ***Deaths*** |  |  |  |
| Total | 1’459,0 | 1’380,0 | 79,0 |
| Per 1,000 patients | 278,6 | 263,6 | 15,1 |

***Table A. Estimated CHD events, strokes, deaths, and events averted following implementation of the CARDIO approach in, Dakar under alternative assumptions for a 2-year implementation phase and a 10-year projection phase.*** Event projections for “No CARDIO” assume control rates consistent with baseline control rates (7%). “CARDIO” assumes control rates equivalent to those measured in the last quarter of CARDIO implementation (19%). Control rates were assumed to remain consistent throughout the projection period.

|  |  |
| --- | --- |
| **Total cost savings from events averted (USD)** |  |
| CHD (implementation phase) | $633’757 |
| CHD (projection phase) | $40’602 |
| Stroke (implementation phase) | $629’209 |
| Stroke (projection phase) | $60’682 |
|  |  |
| **Costs (USD)** |  |
| Incremental Cost | $573’637 |
|  |  |
| **QALYs** |  |
| Incremental QALYs | $265 |
| Incremental QALYs/1,000 patients | $51 |
|  |  |
| **Cost-effectiveness (USD)** |  |
| ICER (per QALY gained) | $2’165 |
| ICER threshold (per QALY gained) ^+^ | $73-$1,166 / QALY |
| National-level GDP per capita in 2018^*^ | 1,458 |
| GDP-based ICER threshold (WHO-CHOICE)^$^ | 4,374 |
| Cost-effective | No |
| Breakeven | 10 |

***Table B. The incremental cost summarizes the difference in total costs (program cost and CV-event costs) for No CARDIO vs CARDIO case.*** +Estimates extracted from [1]. The thresholds are adjusted for PPP and in-country opportunity costs. *Estimates extracted from [2]. $WHO CHOICE stipulates a cost-effectiveness threshold of three times the per-capita GDP

**References**

1. Woods B, Revill P, Sculpher M, Claxton K. Country-level cost-effectiveness thresholds: initial estimates and the need for further research. Value in Health. 2016;19(8):929-35.

2. World Bank. DataBank: World development indicators 2022 [cited 2022 11.03.2022]. Available from: <https://databank.worldbank.org/source/world-development-indicators>.
