# Supplementary material for: Population health impact and economic evaluation of the CARDIO4Cities approach to improve urban hypertension management
Source: PLOS Glob Public Health. 2023 Apr 11;3(4):e0001480. doi: 10.1371/journal.pgph.0001480 (PMC10089359; doi:10.1371/journal.pgph.0001480)
Supplement: Attachment — Submitted filename: 1 Response to reviewers_ Plos Global Public Health_Final.docx [file pgph.0001480.s006.docx]

**Manuscript PGPH-D-22-01401**

**Response to Reviewers**

# Journal requirements:

1. Please amend your detailed Financial Disclosure statement. This is published with the article. It must therefore be completed in full sentences and contain the exact wording you wish to be published.

a. Please clarify all sources of funding (financial or material support) for your study. List the grants (with grant number) or organizations (with url) that supported your study, including funding received from your institution. 
b. State the initials, alongside each funding source, of each author to receive each grant.
c. State what role the funders took in the study. If the funders had no role in your study, please state: “The funders had no role in study design, data collection and analysis, decision to publish, or preparation of the manuscript.”
d. If any authors received a salary from any of your funders, please state which authors and which funders.

The financial disclosure statement has been amended as follows:

*“The Novartis Foundation was the funder of the initiative, oversaw its implementation and data collection, designed the study, planned the publication and prepared the manuscript. Authors SDR, TR, JBo and AAe are employed by Novartis Foundation. Novartis Foundation further provided direct funding to IntraHealth International (author: JBa), Instituto Tellus (MS), Onom Foundation (ND) and the Mongolian Public Health Professionals' Association, MPHPA (EB) to implement the activities of the CARDIO4Cities approach. YC Baxter (YB) received direct funding for consulting and coordinating the CARDIO4Cities activities in São Paulo on behalf of Novartis Foundation. IQVIA (RWO) received funding to collect data on the hypertension care cascade in São Paulo. Novartis Healthcare Pvt. Ltd., Hyderabad, India (GP and ASr) received funding to conduct the economic analysis. The American Heart Association (LV, ASa, JF and LM) was a recipient of funding from the Novartis Foundation for technical assistance and resources to support professional education, quality improvement, monitoring, and evaluation.*

2. We ask that a manuscript source file is provided at Revision. Please upload your manuscript file as a .doc, .docx, .rtf or .tex.

A .docx file was included as requested.

# Reviewers’ comments

**Comments to the Author**
. Have the authors made all data underlying the findings in their manuscript fully available (please refer to the Data Availability Statement at the start of the manuscript PDF file)?

The [PLOS Data policy](https://journals.plos.org/globalpublichealth/s/data-availability) requires authors to make all data underlying the findings described in their manuscript fully available without restriction, with rare exception. The data should be provided as part of the manuscript or its supporting information, or deposited to a public repository. For example, in addition to summary statistics, the data points behind means, medians and variance measures should be available. If there are restrictions on publicly sharing data—e.g. participant privacy or use of data from a third party—those must be specified.

Reviewer #1: No

Reviewer #2: No

The full model including all input data is contained in the supplementary material.

Kindly note that the model relies on population-level summary statistics, which are shared alongside the full computational model. The sharing of individual-level data is not possible due to the restriction of local approvals and ethical committees. Only the local measurement and evaluation partners were granted approval to store and analyze individual-level data and primary data was stored and analyzed locally for all cities. Only aggregated and de-identified data was shared with global partners.

# Reviewer comments to the Author

## Reviewer #1:

This is well-written article concerning the economic evaluation over specific urban populations’ health, resulting from a hypertension management program under the CARDIO4Cities approach. In this sense, this is rather the second, economic evaluation part of the survey and not strictly speaking a scientific paper concerning hypertension management, which would demand much more information, related references and cross-discussion. The fact that it is the economic evaluation of the CARDIO4Cities approach has to be clearly stated to the title and thorough the text.

We thank the reviewer for his kind words and for his critical eye on our manuscriptWe have added the name of the approach to the title (now: “Population health impact and economic evaluation of the CARDIO4Cities approach to improve urban hypertension management) to improve clarity in the framing. We also emphasize the following text in the abstract (lines 41-42):

*“Here, we model the long-term population health impact and cost-effectiveness of a multisectoral urban population health initiative designed to reduce hypertension”*

And in lines 115-117:

*“Here, we expand from those previously reported health improvements and* ***provide an evaluation of the CARDIO4Cities approach’s population health and economic impact****. In this study, we estimate the number of CV events (CHD and stroke) averted over time, and evaluate the approach’s cost-effectiveness across three geographies. “*

As this study is the expansion of a previously published work in order to support the existing approach, there is no discussion concerning the screening criteria for hypertension, which not only are different between the three cities, but are also different throughout the world. Moreover, major discrepancy, somehow stated, concerns the measurement procedure of pressure. Which are the measurement protocols? It is widely known that even if they do exist they are not always followed. On the other hand, when the measurements are performed with electronic pressure meters an overestimation is common. What about some procedures to ensure the proper function of the devices.

The initiative standardized approaches to care and implemented active screening of all patients presenting to primary care facilities. We therefore believe that the screening approach should be comparable and will have had little effect on the results. Nonetheless, we acknowledge the potential bias resulting from the different hypertension definitions. We have therefore added the following clarifying section in the discussion (lines 415-426):

*“**BP measurement and HTN screening practices vary globally. As the first step of the clinical cascade of care, screening coverage can greatly influence the impact of any population health effort. To minimize this variation, CARDIO4Cities included the development of standard screening guidelines that adhere to WHO standards and their integration with existing care protocols. These guidelines include for example, requirements for diagnostic devices and methods (e.g. resting before measurement, patient must be seated). Adherence to guidelines was emphasized through repeated trainings of health care professionals. To maximize screening coverage, active screening was implemented complementary to routine practice. In all three cities, all patients entering primary health units were offered to have their BP measured and were subsequently followed-up depending on BP levels. Further, community outreach events were conducted to integrate asymptomatic healthy, prospective hypertension patients into care. Differences in HTN diagnosis with more stringent thresholds in Ulaanbaatar (130/80 mmHg) than in the other cities (140/90 mm Hg) mean that patients diagnosed with hypertension in Ulaanbaatar may have been considered “normal” in the other cities, leading to a relative overestimation of the hypertensive population.”*

I know that it is not common in your field to apply uncertainty estimations, nevertheless when all these bias parameters are present sensitivity analysis is not enough. Submitted reference [26] indicates many limitations. Some of them are: i) it was not possible to assess the specific impact of interventions tackling underlying determinants of hypertension, ii) a strictly randomized setup (e.g. a randomized control trial) was not possible, iii) the CARDIO4Cities group was not involved in the generation of the data, iv) there was a lack of in-country consensus on the methodology quality and usefulness for clinical practice.

We agree with the reviewer that the scientific limitations resulting from the integrated, real-world implementation of the CARDIO4Cities approach, cannot be fully accounted for by a simple uncertainty analysis. With the restricted data available for analysis, we are unfortunately not able to conduct more advanced analyses, but we have expanded the limitations section to explicitly highlight the points raised by the reviewer and address the additional uncertainties (lines 426-432)

*Additionally, due to the comprehensiveness of the approach and its implementation in a real-world setting without strict randomization and with continued refinement, it is not possible to attribute the impact of the approach to specific interventions. With the available data, the components of the approach can only be evaluated jointly and the “No CARDIO” scenario used as a comparator in this analysis relies on projections from baseline values. Due to its integrated implementation within ongoing care processes, the CARDIO4Cities group was also not directly involved in the generation of the data.*

You have applied a lot of advanced statistical tools to get into the economical aspects of CARDIO4Cities approach, but what about the grounds that it has been set?

We fully agree with the reviewer that the work presented in this manuscript must be seen as a modelling study and mathematical extrapolation based on limited available data. We believe our results provide valuable estimations and complementary evidence to the impact of the CARDIO4Cities approach, in the absence of more robust information. We have clarified this positioning and the call for caution int he discussion (lines 455-459):

*“**With these considerations, we emphasize that our results are a modelling extrapolation on the cost-effectiveness of the CARDIO4Cities approach, based on its early results. Our results are encouraging and provide evidence to the impact and cost-effectiveness of the approach. Yet, real-world data on cardiovascular event rates or health spendings is needed to confirm the robustness of our modelling results.*

We would also like to highlight to the reviewer that reference [26], the key reference on which the modelling study was based, has since been accepted for publication in BMC Public Health. Additional limitations can be found in that reference.

Additionally, major hypertension sources are the living and working conditions, somehow covered by the urbanization notion, but they are much wider. Part of them are the psycho-social issues that have also great importance in the working environment. There are major European organizations dealing with these issues (e.g. EUROFOUND, EU-OSHA).

The aim of the CARDIO4Cities approach was to promote basic, functioning cardiovascular disease prevention and care in low-and middle-income urban settings. We agree with the reviewer that the next step, after these standards of care have been established and implemented, would be to ensure equity and access in cardiovascular care. We have added this as an outlook to the manuscript (lines 401-403).

*“**To ensure that the improved CV population health management reaches all patients, future initiatives should aim to understand the impact of social and broader determinants on health equity.”*

What about GDPR issues of the medical records?

We would like to emphasize that the study at hand was conducted based on population-level summary statistics. Reference 26 contains all required ethics approvals for the primary data collection, and primary data remained under the control of local health authorities at all times.

Finally, I don’t see many non-medication interventions and mainly what about the screening of these non-medication acts. For sure the whole project is based on the intention to protect the health of urban LMICs population, but do they get the same attention as the high income countries?

We have added the global context from the literature to the introduction, based on a scoping review of non-medication interventions against hypertension in LMICs:

Line 89-90:

*“Addressing hypertension at primary care level is one of the most cost-effective methods for minimizing its public health burden (18-20)”*

Lines 96-99:

*“Only 105 studies reported on non-medication public health interventions addressing hypertension in LMICs globally, between 2007 and 2017 (49 of which simultaneously targeted diabetes) (18). The majority neither contained replicable methods, nor reported on the effectiveness or cost-implications of the approach (18).”*

Check out Abstract’s spelling; there are some capitalizations that create bad impression.

We thank the reviewer for supporting us in improving clarity in the manuscript. The capitalizations link to the CARDIO acronym. We have now instead underlined the letters for easier readability.

## Reviewer #2:

It is necessary to explore the reasons for choosing the title of this research and prove it with the latest data for the last 5 years

We thank the reviewer for their critical eye on the methods of data collection and analysis, and thereby strengthening our manuscript. We have changed the title to “Population health impact and economic evaluation of the CARDIO4Cities approach to improve urban hypertension management”, as the main body of our work is a modelling study to estimate the population health and economic impact of the CARDIO4Cities approach.

We agree with the reviewer that additional data would be needed to improve the robustness and reliability of our findings. As we rely on previously published population-level summary statistics for this analysis and no further data of this nature are available, we are unfortunately not in a position to verify the accuracy of the modelling results. We have expanded on the data limitations section in our discussion, and have explicitly added the following consideration in lines 455-459:

*“**With these considerations, we emphasize that our results are a modelling extrapolation on the cost-effectiveness of the CARDIO4Cities approach, based on its early results. Our results are encouraging and provide evidence to the impact and cost-effectiveness of the approach. Yet, real-world data on cardiovascular event rates or health spendings is needed to confirm the robustness of our modelling results.”*

Method. A more comprehensive analysis is needed, namely bivariate and multivariate analysis.

The model currently considers age, sex, blood pressure, and a person’s cardiovascular control status. We agree with the reviewer that more complex analyses would be desirable. Unfortunately, the data required for these are not available. We have described this limitation in the discussion (lines 432-434):

*“Individual-level data or data on potentially confounding variables such as adherence to treatment or co-morbidities were not available, leading us to base our model on population-level characteristics.”*

Discussion. need to be discussed further. not just copying the results

have critically reviewed our discussion section and after the revisions we made, it now contains the following sections:

- Lines 352-363 provide relevance and context to the research
- Lines 364-376 restate the key findings from the results section
- Lines 376-390 discuss possible explanations for the key findings, pertaining to information on the health system and the implementation of the initiative.
- Lines 391-403 discuss the importance of CARDIO4Cities as a scalable approach to improving CV population health globally and to narrowing health inequities.
- Lines 404-411 highlight some of the key requirements from health system for successful implementation of such population heath approaches.
- Lines 412-459 provide an in-depth discussion of the limitations of our work

conclusion. no conclusions were found in the articles written

The conclusion section has been made explicit in lines 461-467:

*“**Overall, our results demonstrate that reducing CVD morbidity and mortality by improving hypertension control at population level is both clinically impactful and broadly economically cost-effective once enough patients are involved. Most importantly, due to the direct relationship between blood pressure and the risk of CV events, improvements in population health can be generated rapidly, even after short intervention periods. The estimated impact of the CARDIO4Cities approach further reflects the potential of public private sector collaborations and simplified approaches that hold patients and health workers at the center, where data is integrated to continuously monitor progress and guide the population health interventions.”*

bibliography. not according to the guide

The revised manuscript now follows the “Vancouver” referencing style, as is used by PLOS. We adapted the references to this style by using the appropriate template in Endnote reference manager.
