# Supplementary material for: Population health impact and economic evaluation of the CARDIO4Cities approach to improve urban hypertension management
Source: PLOS Glob Public Health. 2023 Apr 11;3(4):e0001480. doi: 10.1371/journal.pgph.0001480 (PMC10089359; doi:10.1371/journal.pgph.0001480)
Supplement: Attachment — Submitted filename: 1 Response to reviewers_ Plos Global Public Health_Rev2.docx [file pgph.0001480.s007.docx]

**Manuscript PGPH-D-22-01401**

**Response to Reviewers**

# Journal requirements:

1. Please review your reference list to ensure that it is complete and correct. If you have cited papers that have been retracted, please include the rationale for doing so in the manuscript text, or remove these references and replace them with relevant current references. Any changes to the reference list should be mentioned in the rebuttal letter that accompanies your revised manuscript. If you need to cite a retracted article, indicate the article’s retracted status in the References list and also include a citation and full reference for the retraction notice.

We double checked the reference list and updated references where needed.

2. We have noticed that you have uploaded Supporting Information files, but you have not included a list of legends. Please add a full list of legends for your Supporting Information files after the references list.

Added

# Reviewer comments to the Author

## Reviewer #1:

This is a much more improved version of the manuscript. The majority of the requests have been met clarifying the scope, the limitations and the alternative approaches. Nevertheless, the reported screening guidelines that are reported to be developed by CARDIO4Cities, according to WHO standards, in order to access BP are not included in the submission. In this sense, an extra limitation concerning procedures to ensure the proper function of the screening devices should be added (e.g. specifications, calibration).

We thank the reviewer for his positive feedback to our changes and are grateful for the continued support in strengthening our manuscript. We have elaborated on the screening guidelines and limitations in their execution in the discussion:

417-434 in the discussion.

*To minimize this variation, CARDIO4Cities included the development of standard screening guidelines in each city in collaboration with local authorities and medical societies. These guidelines were integrated into official care protocols. They adhered to standards of the WHO and the International Society of Hypertension (ISH) and included requirements for validated measurement devices and methods, e.g., checking for behavior that could influence BP (recent physical activity, smoking, alcohol or food consumption), ensuring the patient rests for 3-5 minutes before measurement and is seated during measurement, and taking a minimum of three measurements per appointment, at 1 minute intervals, with an arm cuff at heart level. The initiative was fully integrated into ongoing health system processes and relied on local ownership. Therefore, adherence to guidelines was not independently audited by the initiative, limiting its ability to control for potentially confounding external influences. For example, the procurement of BP measurement devices within standard local operating procedures was in part supported by the initiative (e.g., Screening corners in São Paulo, see Table S1). Validation and maintenance of the devices was addressed in official care protocols (e.g., in São Paulo: guidance for annual validation and calibration of measuring cuffs). Yet, responsibility for execution remained with the local health system. Similarly, adherence to care guidelines was emphasized through repeated trainings of health care professionals, and regular quality reviews were established, but all processes were locally driven. We do not foresee a systematic or intentional deviation from the guidelines in any of the participating cities, but differences in the implementation of clinical practice are possible and could bias our results.*

Moreover in lines 89-90 the notion ‘including non-medication interventions’ after ‘primary care level’ should be included.

The clarification has been added (l. 89-90):

*Addressing hypertension at primary care level, including non-medication interventions, is one of the most cost-effective methods for minimizing its public health burden.*

Some linguistic and spelling check will benefit the text.

The lead authors (one of whom is a native English speaker) re-read the full manuscript to ensure it is coherent and written in correct English. In addition, the lead authors used the Editor under the Microsoft Word Proofing functions to check the spelling and grammar.
